# Cold water and harmful algal blooms linked to coral reef collapse in the Eastern Tropical Pacific

**DOI:** 10.7717/peerj.14081

**Published:** 2022-09-28

**Authors:** Caroline Palmer, Carlos Jimenez, Giovanni Bassey, Eleazar Ruiz, Tatiana Villalobos Cubero, Maria Marta Chavarria Diaz, Xavier A. Harrison, Robert Puschendorf

**Affiliations:** 1 School of Biological and Marine Sciences, University of Plymouth, University of Plymouth, Devon, United Kingdom; 2 Seeking Survivors, Yelverton, Devon, United Kingdom; 3 Enalia Physis Environmental Research Centre (ENALIA), Nicosia, Cyprus; 4 Energy, Environment and Water Research Center, The Cyprus Institute, Nicosia, Cyprus; 5 Universidad de Costa Rica, Liberia, Costa Rica; 6 Centro de Investigación en Ciencias del Mar y Limnología (CIMAR), Universidad de Costa Rica, San Jose, Costa Rica; 7 Raising Coral Costa Rica, San Pedro, Costa Rica; 8 Programa de Investigación, Área de Conservación Guanacaste, Liberia, Costa Rica; 9 Centre for Ecology & Conservation, University of Exeter, Penryn, Cornwall, United Kingdom

**Keywords:** Marginal reef, Costa Rica, Immunity, Climate change, Restoration, Upwelling, Resilience-based management

## Abstract

**Background:**

With conventional coral reef conservation methods proving ineffective against intensifying climate change, efforts have focussed on augmenting coral tolerance to warmer water—the primary driver of coral declines. We document coral cover and composition in relation to sea surface temperature (SST) over 25-years, of six marginal reefs in an upwelling area of Costa Rica’s Eastern Tropical Pacific.

**Methods:**

Using reef survey data and sea surface temperature (SST) dating back over 25-years, we document coral cover and composition of six marginal reefs in an upwelling area of Costa Rica’s Eastern Tropical Pacific in relation to thermal highs and lows.

**Results.:**

A ubiquitous and catastrophic coral die-off event occurred in 2009, driven by SST minima and likely by the presence of extreme harmful algal blooms. Coral cover was dramatically reduced and coral composition shifted from dominant branching *Pocillopora* to massive *Pavona*, *Porites*, and *Gardineroseris*. The lack of coral recovery in the decade since indicates a breach in ecosystem tipping-point and highlights a need for resilience-based management (RBM) and restoration. We propose a locally tailored and globally scalable approach to coral reef declines that is founded in RBM and informed by coral health dynamics.

## Introduction

Climate change-induced marine heatwaves are driving coral mass mortality on iconic reefs (Frölicher & Laufkötter, 2018; Hughes et al., 2017). With conventional coral reef management strategies no longer effective against climate change (Mcleod et al., 2019), technological developments and interventions are targeted at improving coral resilience to warmer water (Anthony et al., 2017; Camp, Schoepf & Suggett, 2018; Oppen et al., 2017). The heterogeneity and diversity of coral reefs, with climate change and local impacts (Mcleod et al., 2019), however, suggests that conservation strategies focussing solely on resilience to one factor, will be neither sufficient nor broadly scalable (Bauman et al., 2010; Dubinksy & Stambler, 1996). Instead, working to understand local coral health threats and forming evidence-based management strategies and priorities may enable more effective and ubiquitous coral conservation and restoration (Mcleod et al., 2019).

While the cause of coral declines does not impact recovery potential (Graham, Nash & Kool, 2011), it will likely influence the choice and efficacy of management strategies. How to increase resilience of species and ecosystems under threat by direct and indirect human impacts is now a fundamental question in conservation biology (Folke et al., 2010). Resilience-based management (RBM) proposes an integrative approach to fosters stress tolerance, promote recovery and facilitates adaptation of both the target ecosystem and society (Mcleod et al., 2019). For efficacy however, RBM and associated policies must be supported by locally-relevant research outcomes, such as reef site species diversity, functional group identification and risk assessment, and include identifying stressors responsible for driving coral declines or promoting reef resilience (Mcleod et al., 2019). While such parameters have been studied for reefs within the core tropical coral distribution, factors influencing the health of marginal reefs have only recently emerged as relevant for stemming global coral declines (Burt et al., 2020).

Marginal coral reefs, such as those of the Eastern Tropical Pacific (ETP) (Glynn et al., 2017), exist at the periphery of reef-building coral’s environmental tolerance range (Guinotte, Buddemeier & Kleypas, 2003). Reef development in the ETP is influenced by particular environmental factors operating in the region (Toth, Macintyre & Aronson, 2017). For example, in response to turbidity and exposure to oceanic influence, the majority of the ETP reef framework accretion is in shallow waters and the small and discontinuous reefs are usually sheltered from direct exposure to seasonal upwellings (Glynn & Ault, 2000; Glynn, Manzello & Enochs, 2016; Toth, Macintyre & Aronson, 2017). In contrast to other coral reef provinces, ETP reefs lack a definite zonation, their communities have a few coral species (47 in the entire ETP), and the number of reef building genera is low (four out of 11), and includes branching *Pocillopora*, and massive *Pavona*, *Porites*, and *Gardineroseris*. Coastal areas prone to high sediment loads, freshwater influxes and restricted water circulation might show limited reef development by the four genera (Glynn, Manzello & Enochs, 2016). Oceanic islands and transitional areas with marked seasonality and marginal exposure to upwelling tend to exhibit best-developed reefs at greater depths, mostly by the three genera of massive species. It is nevertheless noteworthy that despite the marginal character of ETP reefs (Toth, Macintyre & Aronson, 2017), structurally simple and with a low number of species, they have biological and ecological interactions as complex as coral reefs in other coral provinces (Enochs & Glynn, 2017; Glynn, Manzello & Enochs, 2016).

Locally adapted to conditions considered sub-optimal, marginal reefs have been proposed as possible refugia from marine heatwaves (Camp, Schoepf & Suggett, 2018; Glynn, 1996; Rixen, Jiménez & Cortés, 2012; Romero-Torres et al., 2020). Refugia status, however, does not guarantee protection nor sustainability (Chollett, Mumby & Cortés, 2010; Freeman, 2015) and the cornerstones of reef resilience may well differ from those of other reefs (Nyström et al., 2008), especially in the context of global climate change and more extreme weather events which are having an impact on coastal upwelling intensity (Bakun, 1990). Extremes in cold water intrusion are known to be a tropical coral stressor and have caused coral die-offs in more seasonal systems (Roberts et al., 1982).

The marginal coral communities of the Papagayo region on the Pacific coast of Costa Rica experience extreme environmental shifts with seasonal upwellings (Glynn et al., 2017). North-northeast trade winds displace surface waters between November and April, drawing-up cold (Alfaro et al., 2012; Jiménez, 2001), nutrient-rich (Stuhldreier et al., 2015a), hypoxic (Karstensen, Stramma & Visbeck, 2008) and acidic (Rixen, Jiménez & Cortés, 2012; Sánchez-Noguera et al., 2018) oceanic water to shallow coral communities. The apparent physiological tolerance of these marginal corals to shifting environmental conditions seems particularly pertinent as scientists race to find traits that increase coral tolerance (van Oppen et al., 2017).

While Costa Rica’s marginal coral communities have historically experienced El Niño-associated mass bleaching events (Glynn, 1984, 1990; Jiménez, 2001), corals in the Papagayo region were not severely affected by the record-breaking 1997–1998 global bleaching event (Jiménez et al., 2001; Wilkinson, 1999). Cold water intrusions, upwellings (Sánchez-Noguera et al., 2018) and high cloud-cover (Jimenez, 2002) most likely locally off-set the marine heatwave (Jiménez et al., 2001), and supports these marginal reefs as coral refugia. Coral reefs most resilient to system collapse and phase shifts, however, have high biodiversity, giving them a larger pool of species to maintain ecosystem functions (Oliver et al., 2015), spatial heterogeneity and connectivity (Nyström et al., 2008), which are not characteristics of ETP coral communities (Glynn et al., 2017).

Coral reefs are threatened by other climate change-induced environmental shifts, beyond marine heatwaves, and local impacts, which need to be considered for effective conservation (Mcleod et al., 2019). While harmful algal blooms (HABs) typically coincide with natural influxes of nutrients during upwelling events (Bakun et al., 2015), proliferating coastal development has increased their frequency and intensity globally (Lapointe et al., 2019). HABs, historically a natural, short-lived and sporadic occurrence in Papagayo have increased (Morales-Ramírez et al., 2001) and compromise coral health (Anderson, 1997; Cortés & Jimenez, 2003; Guzmán et al., 1990).

We are aware that coral reefs in Costa Rica, like elsewhere have declined, and especially in the system we describe here some of these impacts have been previously noted (Jiménez et al., 2001). However existing data had not yet been updated to note further shifts in local coral status and analysed to test and quantify the magnitude of these population declines across space and time. Here, we analyse a coral reef dataset spanning over the 25-year period between 1994 to 2019. We model for temporal changes in coral cover and genera diversity at six reef sites in relation to sea surface temperature (SST). We highlight the drivers of a mass coral die-off and discuss the implications for the development of effective conservation and restoration strategies.

## Materials and Methods

### Study system

The region of Papagayo is under the influence of wind-driven coastal upwelling particularly during the dry season (December–April). However, off-season upwellings are common and seawater conditions are similar to those during the upwelling season at least for a few days (Rixen, Jiménez & Cortés, 2012). The strong seasonality is clearly manifested in the mean (±SD) values during the seasons (dry and wet season). For seawater parameters in the study area the following has been recorded: temperature (22.2 ± 1.7 and 30.08 ± 0.27 °C, 1.5 m depth), pH (7.90 ± 0.12 and 8.02 ± 0.03), salinity (34.9 ± 0.8 and 32.7 ± 1.5 psu), and chlorophyll-*a* (1.19 ± 0.66 and 0.43 ± 2.88 mg m^−3^), (Alfaro et al., 2012; Jiménez et al., 2010; Sánchez-Noguera et al., 2018; Saravia-Arguedas et al., 2021). Physiological processes, such as calcification and respiration, of corals and other calcifying organisms are affected by these seasonal changes (Rixen, Jiménez & Cortés, 2012; Sánchez-Noguera et al., 2018).

Papagayo is the coastal region in the north-western Pacific of Costa Rica that until recently exhibited profuse coral communities and numerous small and discontinuous reefs in sheltered areas of islands, islets and coves (Cortés & Jimenez, 2003; Glynn et al., 2017; Jiménez et al., 2010). Most of the reef building was done by massive colonies of *Pavona* spp., *Gardineroseris planulata* and branching species of *Pocillopora*. The latter were responsible for significant framework accretion in a few shallow (<8 m depth) places along the Papagayo coast (Cortés & Jimenez, 2003; Glynn, Druffel & Dunbar, 1983; Toth, Macintyre & Aronson, 2017).

The strong seasonality of the area and the absence of major rivers, and, hence, year-round fresh water input producing low salinity conditions and high sediment loads, offered more suitable habitats for coral reef and communities’ development than other localities in the Pacific of Costa Rica (Jiménez et al., 2010) and partially explain the prevalence of two coral genera (*Pocillopora* and *Porites*) as the main reef builders in southwestern Pacific Costa Rica (Glynn et al., 2017). The biodiversity of corals in the northern Papagayo area, 22 zooxanthellate species, is the highest of mainland Costa Rica (last tally in Glynn et al. (2017)). Seventeen of those species are found in the study area of Islas Murciélago, making it one of the most important areas of the country in terms of biodiversity of coral and coral reef-associated species, and coral habitats.

### Coral reef monitoring

**Figure 1:**
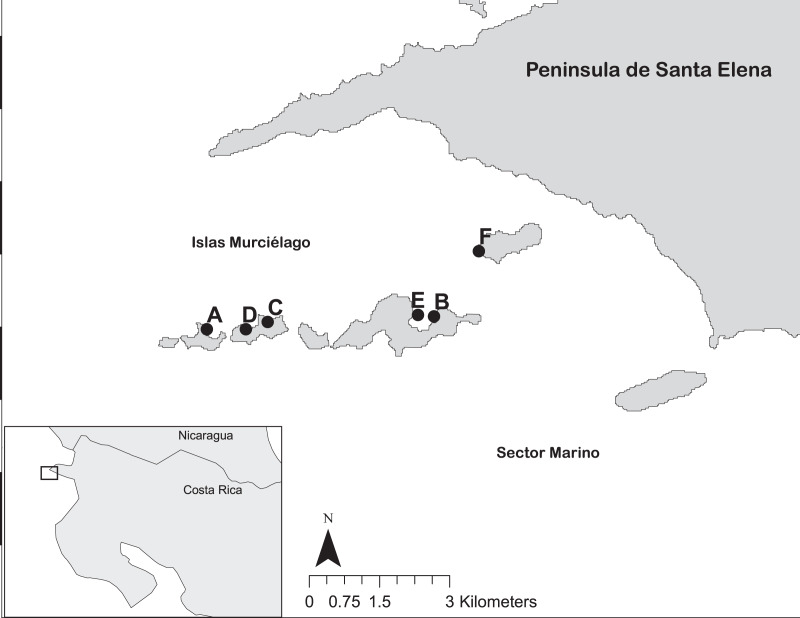
The reef sites at Islas Murciélago and Sector Marino of Área de Conservación Guanacaste (ACG), within Golfo de Papagayo of Costa Rica. Numbered dots are reef sites: A = San Pedrito, B = Cocinera, C = Golondrina, D = Lara, E = Dos Emes, F = Pelada.

Coral reef surveys were conducted at Islas Murciélago, Área de Conservación Guanacaste’s (ACG) Marine Protected Area, Papagayo, Costa Rica (10°N 86°W; Fig. 1) between 1994 and 2019. Ten-metre line intercept transects were used to measure live coral cover (to the nearest 5 cm) and record coral genera at six sites; Isla San Pedrito, Cocinera, Lara, Golondrina, Dos Emes and Pelada (Fig. 1). In total, 732 transects were conducted between three and 12 m deep in replicates of three to five (Table S1). Monthly sea surface temperatures (SST) from 1982 to 2019 were obtained from NOAA OI.v2 (Reynolds et al., 2002) for Papagayo (1 × 1 grid centred 10°N 86°W), and upper and lower coral bleaching thresholds were defined as 1 °C above or below the mean monthly SST (Donner et al., 2005). Harmful algal bloom (HABs) observations in the Papagayo region were recorded by CJ and MMCH.

Fieldwork were approved by the Área de Conservación Guanacaste (project number FOI-004-001).

### Data analysis

Statistical analysis was conducted in R v3.6.3 (Team, 2020) (Supplemental Material). We used linear mixed models (LMMs) in R package *lme4* (Bates et al., 2015) to model coral cover dynamics over time at each site. Logit-transformed proportion coral cover was fitted as a response variable with a Gaussian error structure (Warton & Hui, 2011), and year as a predictor. We included depth and transect ID as random intercept terms, allowing us to marginalise the effects of each when estimating the average effect of year for each site. We evaluated support for both linear and quadratic effects of year by comparing models with the AIC. Models containing the quadratic term for year had highest support in the data for all sites (Table S2A). We generated posterior mean predictions and 95% credible intervals for each site-specific best-supported model in the R package *brms* (Bürkner, 2017, 2018) (Table S2B).

To examine the drivers of coral declines, we fitted bivariate response mixed effects models in *brms*. Each model contained a 2-column response of a SST variable (minimum or maximum) and logit-transformed mean coral cover per year-site combination, and linear year as a predictor variable. These models allow us to control for temporal trends in each response whilst simultaneously estimating the posterior correlation among the residuals from each model. Therefore, if there is a true association relationship between SST and coral cover, positive residuals in one model should drive a consistent directional relationship in the residuals of the other, inducing a correlation (see Houslay & Wilson, 2017). If maximum SST drives coral declines, a negative correlation among residuals would be expected (positive residuals representing above average SST drive negative residuals in the coral model representing greater than average loss of coral cover). The converse would be the case if minimum SST drives coral loss, with a positive correlation expected. We tested for this relationship between coral cover and *current year* SST_max_ and SST_min_, as well as lagged models incorporating SST data from the year prior to coral cover surveys, referred to as lag+1 model. We evaluated the significance of the posterior residual correlations based on whether the credible intervals for the estimate crossed zero.

## Results

**Figure 2:**
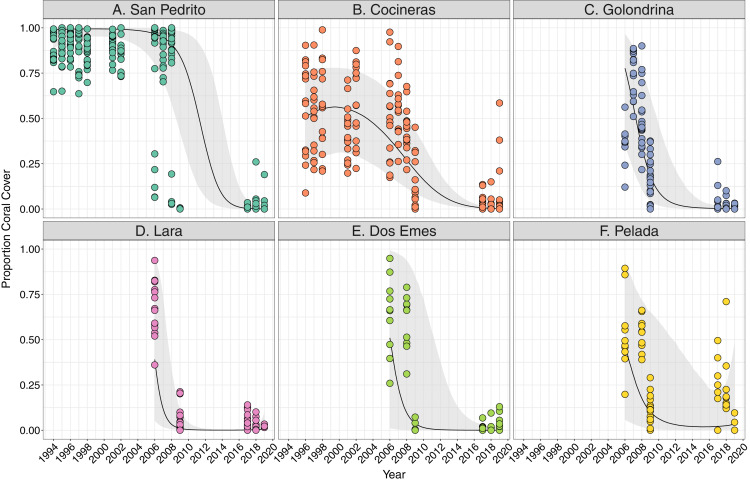
Trends in coral cover across six sites at Islas Murciélago, Costa Rica. Points are raw survey data. Lines and shaded areas represent means and standard errors of a linear mixed effects model examining coral cover dynamics that marginalise depth and transect variation. Model estimates and model selection results are presented in Table S1.

Coral cover varied both among and within the six sites between 1994 and 2018, and all showed a significant decline overall (Fig. 2). Coral cover declines were greatest between 2008 and 2009 at all sites (Fig. 2), ranging between an approximate 20% decrease in coral cover at Cocinera to 100% at San Pedrito (Fig. 2). Rates of coral cover declines differed among sites (Tables S2A, S2B), with Pelada, Lara and San Pedrito having the sharpest decreases (Fig. 2). The most significant decrease in coral cover occurred between October 2008 and May 2009 surveys. Pelada had coral cover recovery to pre-2009 levels (Fig. 2).

**Figure 3:**
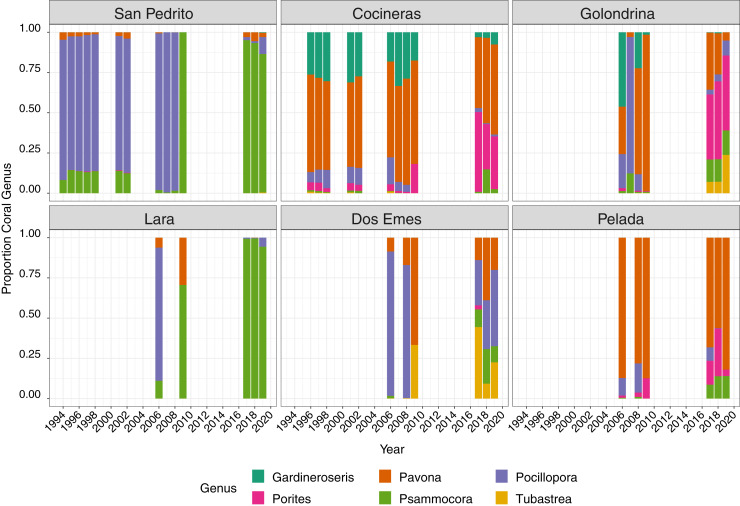
Mean proportion of each coral genera between 1994 and 2019 across six sites.

Six coral genera were recorded at the sites; *Pocillopora, Pavona, Porites, Gardineroseris, Psammocora* and *Tubastraea*, the mean proportion of which varied through time and among site, with a community structure shift after 2009 (Fig. 3). Between 1996 and 2008, *Pocillopora* comprised approximately 50% of corals at all sites (Fig. 3), *Pavona* 25% and *Gardineroseris*, *Psammocora* and *Porites* collectively accounting for the remaining 25%. In 2009, no live *Pocillopora* was recorded and *Pavona* contributed the greatest proportion (40%) followed in decreasing order, by *Psammocora*, *Porites*, *Gardineroseris* and *Tubastraea* (Fig. 3). By 2017, all six coral genera were again present at Islas Murciélago, though in differing proportions to pre-2009 (Fig. 3).

**Figure 4:**
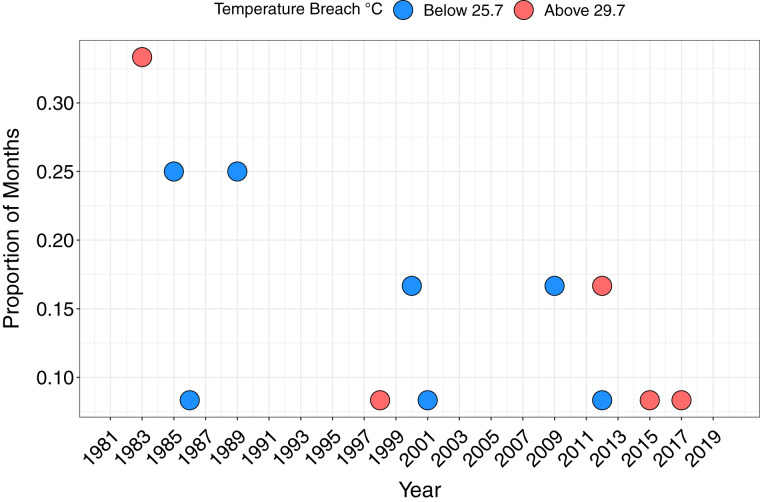
The proportion of months per year breaching the lower and upper temperature thresholds. Breaching the lower (below 25.7 °C) and upper (above 29.7 °C) temperature thresholds, defined as 1 °C above or below the maximum and minimum mean monthly SST, respectively, based on monthly mean SST.

The maximum monthly mean SST for Papagayo was 28.7 °C between 1982 and 2018, and minimum monthly mean 26.7 °C. The upper thermal bleaching threshold was breached in five years; 1983, 1998, 2012, 2015 and 2017, and the lower thermal bleaching threshold breached in seven years, including consecutive months of February and March 2009 (Fig. 4). Short-lived and sporadic HABs were recorded within Papagayo up to 2006, with a continuous series of HABs during the 2008–2009 upwelling season. Up to monthly HABs were observed between 2012 and 2017 (Supplemental Figures).

**Figure 5:**
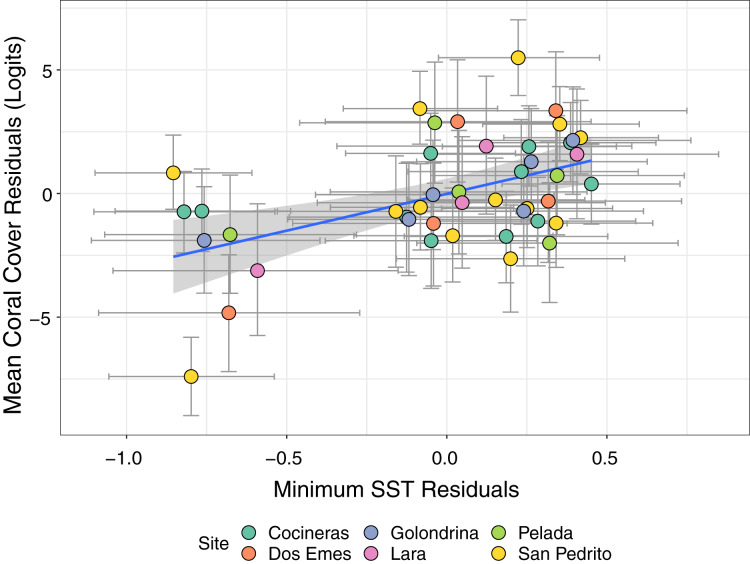
Estimates from a bivariate mixed effects model examining the posterior correlation between annual minimum sea surface temperature (SST) and proportion of coral cover. We detected a significant positive correlation between the residuals of the SST and coral models, indicating that colder than average minimum SST (negative residuals on x axis) are associated with accelerated loss of coral cover (negative residuals on y axis). Points are coloured by site.

Bivariate response modelling revealed a significant positive correlation between minimum SST and magnitude of coral declines (posterior mean 0.47; 95% credible interval [0.19–0.7]; Fig. 5). This model explained 17% of variation in SST_min_ (95% credible interval [4.9–31.4%]) and 58.6% of variation in mean coral cover (95% credible interval [43–68%]). Conversely, we detected no significant correlation between maximum SST and coral cover (posterior mean = −0.17; 95% credible intervals [−0.46 to 0.15]; Fig. S1). Similarly, there was no evidence that lag+1 SST_max_ or SST_min_ were associated with coral declines (credible intervals of all posterior correlations crossed zero). Collectively these results show that colder than average current-year minimum SSTs are associated with coral declines.

## Discussion

The global demise of coral reefs stems from their sensitivity to stressors, such as thermal extremes, which breaks down the fundamental symbiosis that guarantees coral health (Douglas, 2003). The demise of large portions to the Great Barrier Reef after sequential thermally-induced bleaching events has been worse than the effects of category five cyclones going over the same area (Dietzel et al., 2021). With similar consequences, but a different stressor, coral declines over the past 25 years at Islas Murciélago, in Costa Rica’s Área de Conservación Guanacaste (ACG) are associated with cold water events and also likely impacted by harmful algal blooms (HAB’s). A significant coral die-off occurred during the 2008–2009 extreme upwelling, which breached the lower thermal threshold and coincided with HABs. The lack of coral cover recovery at five of the six sites in the decade since the die-off suggests ecosystem collapse, with a shift to a low resilience coral-poor altered state (Graham, Nash & Kool, 2011). The lack of influence of marine heatwaves on ACG’s coral cover, challenges the assertion that they are the ubiquitous and most pressing threat to coral reefs (Hughes et al., 2018b). To secure both ecological and socioeconomical functions of ACG and surrounding reefs, resilience-based management (RBM) strategies can be developed from these research outcomes.

### Drivers of mass coral die-off

The cold-water upwelling during La Niña of 2008 to 2009 and a widespread HAB’s event drove a crash in coral cover at Islas Murciélago in ACG. Such an extreme upwelling had not occurred since 2000. During upwellings, rapid temperature drops of up to 10 °C (Rixen, Jiménez & Cortés, 2012) potentially induces coral bleaching and mortality through cold-water shock (Muscatine, Grossman & Doino, 1991), as well as hypoxia, increased acidity and low aragonite saturation state (Sánchez-Noguera et al., 2018). While Islas Murciélago’s coral communities are locally adapted to seasonal upwellings (Stuhldreier et al., 2015a, 2015b), this did not confer reef resilience to the 2009 extreme event, which appeared to breach tolerance thresholds for the majority of corals (Palmer, 2018a). HABs are no longer an infrequent natural occurrence fuelled by the increased primary productivity of cold water upwellings, but a direct result of poor wastewater management, with devastating impacts to marine ecosystems and the fishing communities that depend on them (Lapointe et al., 2019; Paerl & Scott, 2010). The Costa Rican coastal current predominantly brings water from the highly developed southern region to the undeveloped, national park coastline of ACG’s MPA. Likely, the synergy between persistant HABs and the 2009 extreme upwelling caused a devastating impact on Islas Murciélago’s coral communities (Lapointe et al., 2019; Paerl & Scott, 2010).

The lack of relationship between coral declines and SST maxima at Islas Murciélago, suggests corals are able to physiologically tolerate warmer water (Palmer, 2018b). Such physiological tolerance would likely confer ecosystem resilience to the increasing threat of elevated SST (Romero-Torres et al., 2020), provide refugia (Freeman, 2015) and is highly relevant for research into increasing coral warmer water tolerance (*e.g.*, (Anthony et al., 2017; Oppen et al., 2017; Camp, Schoepf & Suggett, 2018)). While levels of coral immunity relate directly to tolerance (Palmer, 2018a; Palmer, Bythell & Willis, 2010), the mechanisms of Islas Murciélago coral physiological tolerance have not been explored. A type II error is also a possibility, with Papagayo mean monthly SST breaching upper thermal bleaching thresholds in 2012 and 2015, when surveys were not conducted. Notably, however, Papagayo did not experience a breach in upper thermal bleaching threshold in several years reported as global SST maxima, including 2005 to 2006, 2009 to 2010 and 2016 (Hughes et al., 2018a), suggesting that local oceanographic and/or atmospheric buffering has protected reefs from marine heatwaves (Jiménez, 2002).

### Coral decline and recovery rates among sites

The rate of coral declines at Islas Murciélago varied among the six reef sites, with those dominated by the highly susceptible coral genus, *Pocillopora* (Palmer, Bythell & Willis, 2010) declining faster and recovering less. This primary loss of the most susceptible coral genera (Côté & Darling, 2010), drove a composition shift to massive and encrusting *Pavona* and *Psammocora* spp., at San Pedrito, Lara and Dos Emes, consistent with other reefs during extreme events (Knowlton, 2001). Previous studies have documented significantly lower levels of constituent immunity of fast growing (*e.g. Pocillopora* spp) compared to slower growing massive corals such as *Porites* spp (Palmer, Bythell & Willis, 2010), which tend to invest more in immunity and seem to be more resistant to disease and bleaching given that they are longer lived (Palmer, 2018a). The reduction of benthic structural complexity provided by coral habitats (*e.g*., *Pocillopora* dominated communities and reefs), translates into a loss of potential habitats for associated species and changes in ecological processes affecting the entire ecosystem. Important ecological changes have been documented in the area of Papagayo (Bahia Culebra) after drastic deterioration of coral habitats during the last decade: a reduction of ca. 40% of reef fish orders (Arias-Godínez et al., 2019) changes in the trophic structure of reef fish assemblages (decline of fish mesopredators; Arias-Godínez et al., 2019), and drastic changes in the composition of the biodiversity associated with *Pocillopora* colonies (obligate commensal species replaced by boring and opportunistic or facultative species Salas-Moya et al., 2021).

Such high and non-random coral loss (Côté & Darling, 2010) from all sites is consistent with the low ecosystem resilience reported for low diversity reefs (McClanahan et al., 2012). Similarly, the lack of recovery at five sites indicates that the 2009 die-off event pushed ACG’s coral communities beyond their tipping point, preventing significant recovery and causing system collapse (Nyström et al., 2008).

Coral recovery was hampered by local *Pocillopora* extirpations, which likely reduced larval and fragmented propagule supply required for recruitment (Ayre & Miller, 2004). Furthermore, with acidic upwelled water promoting rapid skeletal dissolution and sea urchin dominance causing intensive bio-erosion (Alvarado et al., 2016), dead coral is rapidly reduced to unconsolidated rubble beds, unsuitable for coral recruitment (Fordyce et al., 2019). In contrast, Pelada regained pre-decline coral cover by 2017, including a low proportion of *Pocillopora*, which may have been enabled by the higher proportion of more tolerant massive corals and site exposure to larvae-carrying ocean currents. Such recovery suggests that, with careful management, ACG’s reefs could recover and continue to support ecological and societal ecosystem services through escalating climatic changes.

## Conclusions

### Resilience-based management for coral reef restoration

The exploration of ACG’s coral reef dynamics provides locally-relevant research outcomes, beneficial for implementing RBM (Mcleod et al., 2019) and which refute the generic narrative that marine heatwaves are the primary driver of declines. Conservation priorities of ACG’s marginal reef system should target preventing further coral loss, aiding its recovery and promoting ecological and social resilience (Mcleod et al., 2019). To prevent further coral loss, improving the regions wastewater management and coastal land use is vital for reducing the frequency and magnitude of HABs. ACG’s limited coral recovery highlights a clear need for coral restoration, as well as substrate consolidation. While restoring reefs with fast-growing *Pocillopora* would facilitate rapid coral cover recovery, the impact of cold events on *Pocillopora-*dominated reefs demonstrates their limited resilience. Slower growing, more tolerant massive coral species (Palmer, Bythell & Willis, 2010) should therefore be included in restoration efforts.

The inevitability of increasing climate-associated extreme events, and the uncertainty of whether these may include warming, cooling, increased hypoxia and acidity (Bakun et al., 2015), supports the need to explore experimental conservation approaches—with any risk dwarfed by the greater risk of not acting (Mcleod et al., 2019). With unknown, multiple and/or variable perturbations affecting corals, beyond marine heatwaves, determining how coral stays healthy, maintains or re-establishes homeostasis and survives a variety of challenges is key for effective conservation (Palmer, 2018a). As such, understanding coral immunology is vital, with the potential recovery, such as re-establishing homeostasis of the meta-organism (holobiont) under new, or perturbed, conditions (Palmer, 2018a, 2018b) dependent on investment in and efficacy of immune mechanisms (Palmer, 2018a; Palmer, Bythell & Willis, 2010). Understanding such fundamental biological knowledge, beyond tolerance of marine heatwaves, is key for developing optimised conservation strategies that can be adapted across all reef systems.

Like many reefs globally, ACG’s coral communities have been pushed beyond their tipping point and are unlikely to recover without proactive, locally targeted conservation interventions. The importance of understanding coral tolerance biology to a range of impacts is highlighted by cold water as the primary driver of ACG’s coral loss and underscored by the uncertainty of future climate perturbations. Locally targeted, evidence-driven RBM approaches that incorporate immunology into coral restoration presents a more viable and globally scalable strategy for conserving coral reefs.

## Supplemental Information

10.7717/peerj.14081/supp-1 Supplemental Information 1 Supplementary material.

10.7717/peerj.14081/supp-2 Supplemental Information 2 Selection of images pre and post declines of reefs in the study system.

10.7717/peerj.14081/supp-3 Supplemental Information 3 Raw data and readme worksheet. The abbreviations used in the worksheet for the analysis

## Additional Information and Declarations

### Field Study Permissions

The following information was supplied relating to field study approvals (*i.e*., approving body and any reference numbers): Fieldwork was approved by the Area de Conservacion Guanacaste (project number FOI-004-001).

### Data Availability

The following information was supplied regarding data availability: The code and data is available at GitHub: https://github.com/xavharrison/CoralDeclines2020.
