# Supplementary material for: Cold water and harmful algal blooms linked to coral reef collapse in the Eastern Tropical Pacific
Source: PeerJ. 2022 Sep 28;10:e14081. doi: 10.7717/peerj.14081 (PMC9526400; doi:10.7717/peerj.14081)
Supplement: Supplemental Information 1 [file peerj-10-14081-s001.docx]

**Table S1.** Total transect number per site per year.


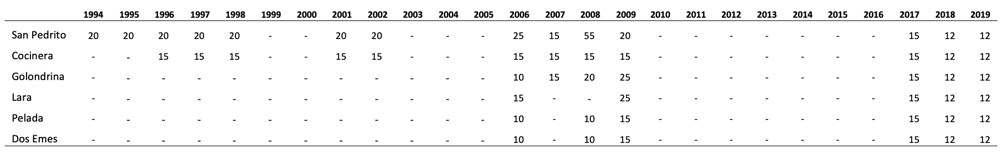


**Table S2. (A)** Model selection results examining relative support for linear or quadratic effects of year using the AIC. Values of 0 indicate the model containing the term in that row is the best supported model. Positive values indicate the change in AIC compared to the top (0) model and represent a loss of predictive power. E.g. for San Pedrito, fitting only a linear Year effect results in dramatic loss of predictive power. **(B)** Model estimates from best supported model for each site, containing means and 95% credible intervals. We also present estimates of variance components of random intercepts for Depth and Transect, presented as standard deviations (mean and 95% credible intervals).

| **(A) AIC Model Selection** | | | | |  |  |
| --- | --- | --- | --- | --- | --- | --- |
| **Model** | **San Perdito** | **Lara** | **Golondrina** | **Cocinera** | **Dos Emes** | **Pelada** |
| Year | 58.06 | 9.36 | 6.95 | 44.3 | 6.28 | 35.06 |
| Year^2^ | 0 | 0 | 0 | 0 | 0 | 0 |
|  |  |  |  |  |  |  |
| **(B) Parameter Values** | | | | |  |  |
|  |  |  |  |  |  |  |
| *Parameter* | *Posterior Mean* | *95% Credible Intervals* | |  |  |  |
| ***San Pedrito*** |  |  |  |  |  |  |
| Intercept | 3.34 | 0.66 - 6.09 | |  |  |  |
| Year | -49.77 | -59.11 - 40.34 | |  |  |  |
| Year^2^ | -30.25 | -38.59 - -22.06 | |  |  |  |
| sd(Transect) | 1.95 | 0.12 - 3.88 | |  |  |  |
| sd(Depth) | 2.5 | 1.21 - 4.7 | |  |  |  |
|  |  |  |  |  |  |  |
| ***Lara*** |  |  |  |  |  |  |
| Intercept | -5.11 | -11.96 - 1.83 | |  |  |  |
| Year | -8.02 | -12.19 - -2.34 | |  |  |  |
| Year^2^ | -8.02 | -12.19 - -2.34 | |  |  |  |
| sd(Transect) | 1.97 | 0.67 - 3.61 | |  |  |  |
| sd(Depth) | 8.25 | 1.59 - 17.55 | |  |  |  |
|  |  |  |  |  |  |  |
| ***Golondrina*** |  |  |  |  |  |  |
| Intercept | -1.99 | -4.38 - 0.33 | |  |  |  |
| Year | -2.96 | -3.73 - -2.21 | |  |  |  |
| Year^2^ |  |  | |  |  |  |
| sd(Transect) | 0.41 | 0.02 - 1.16 | |  |  |  |
| sd(Depth) | 2.31 | 0.79 - 6.07 | |  |  |  |
|  |  |  |  |  |  |  |
| ***Cocinera*** |  |  |  |  |  |  |
| Intercept | -1.3 | -2.54 - -0.21 | |  |  |  |
| Year | -24.87 | -28.58 - -21.02 | |  |  |  |
| Year^2^ | -13.7 | -17.98 - -9.54 | |  |  |  |
| sd(Transect) | 0.25 | 0.01 - 0.84 | |  |  |  |
| sd(Depth) | 1.06 | 0.24 - 3.15 | |  |  |  |
|  |  |  |  |  |  |  |
| ***Does Emes*** |  |  |  |  |  |  |
| Intercept | -5.07 | -10.73 - 0.84 | |  |  |  |
| Year | -28.3 | -33.64 - -23.11 | |  |  |  |
| Year^2^ | -1.01 | -9.65 - 7.66 | |  |  |  |
| sd(Transect) | 0.64 | 0.03 - 1.63 | |  |  |  |
| sd(Depth) | 5.31 | 2.04 - 12.91 | |  |  |  |
|  |  |  |  |  |  |  |
| ***Pelada*** |  |  |  |  |  |  |
| Intercept | -3.04 | -9.63 - 4.22 | |  |  |  |
| Year | -24.87 | 4.47 - 21.77 | |  |  |  |
| Year^2^ | -13.7 | 18.88 - 37 | |  |  |  |
| sd(Transect) | 0.3 | 0.01 - 0.91 | |  |  |  |
| sd(Depth) | 6.54 | 2.61 - 15.89 | |  |  |  |

**Table S3**. Model estimates from a bivariate mixed effects model jointly modelling coral cover and Sea Surface Temperature (SST) as a function of year. Only the model of current year SST minimum produced a significant posterior correlation between SST and coral cover residuals (bold values). Proportion of explained variance (R^2^) was similar for both coral cover and SST for all models.

| **Model** | **Posterior Correlation [95% Credible Interval]** | **Coral R^2^ Mean [95% Credible Interval]** | **SST R^2^ Mean [95% Credible interval]** |
| --- | --- | --- | --- |
| SST Min | ***0.48 [0.18 - 0.69]*** | 0.58 [0.43 - 0.68] | 0.17 [0.05 - 0.32] |
| SST Max | - 0.17 [-0.46 - 0.15] | 0.59 [0.44 - 0.68] | 0.33 [0.16 - 0.48] |
| SST Min Lag+1 | -0.1 [-0.41 - 0.22] | 0.58 [0.43 - 0.68] | 0.19 [0.06 - 0.33] |
| SST Max Lag+1 | -0.03 [-0.35 - 0.29] | 0.59 [0.44-0.68] | 40.7 [0.23 - 0.53] |


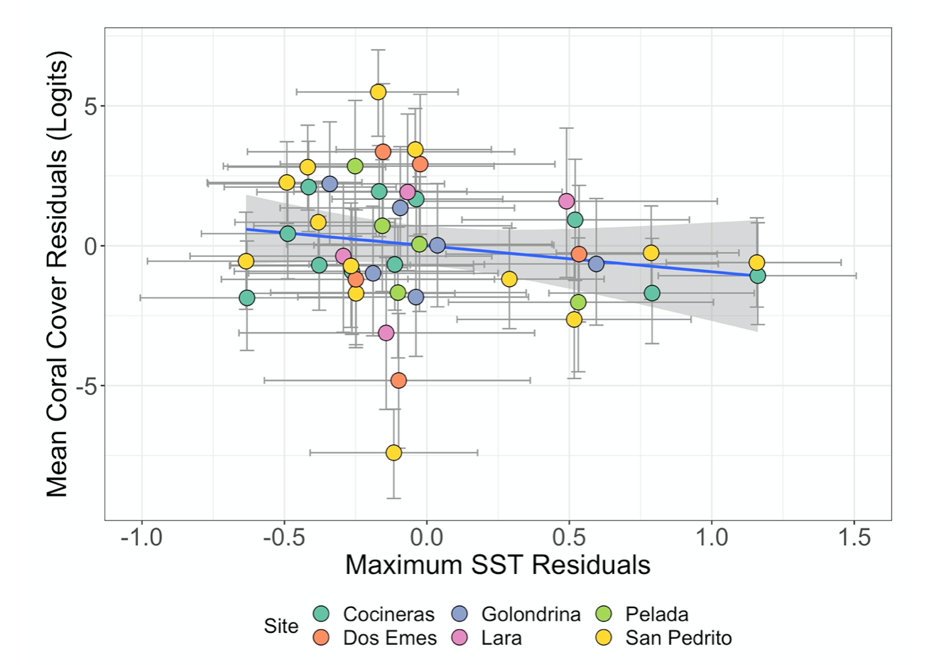


**Figure S1**: Posterior model estimates of a bivariate mixed model estimating the correlation between maximum SST and logit-transformed coral cover. There was no evidence of a significant correlation between these two traits (posterior mean = -0.17 ; 95% credible intervals -0.46 – 0.15). Points are coloured by site.

R code available on R Markdown at <https://github.com/xavharrison/CoralDeclines2020>
