# Supplementary material for: Cold water and harmful algal blooms linked to coral reef collapse in the Eastern Tropical Pacific
Source: PeerJ. 2022 Sep 28;10:e14081. doi: 10.7717/peerj.14081 (PMC9526400; doi:10.7717/peerj.14081)
Supplement: Supplemental Information 2 [file peerj-10-14081-s002.docx]

**Supplementary images, pre and post coral reef collapse, San Pedrito Reef**

San Pedrito Reef pre collapse, images span from 2006-2008.


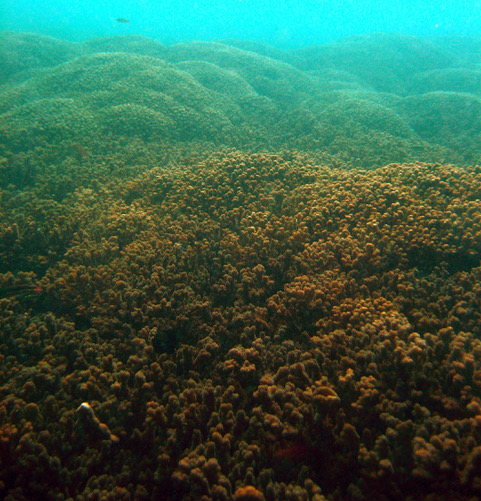


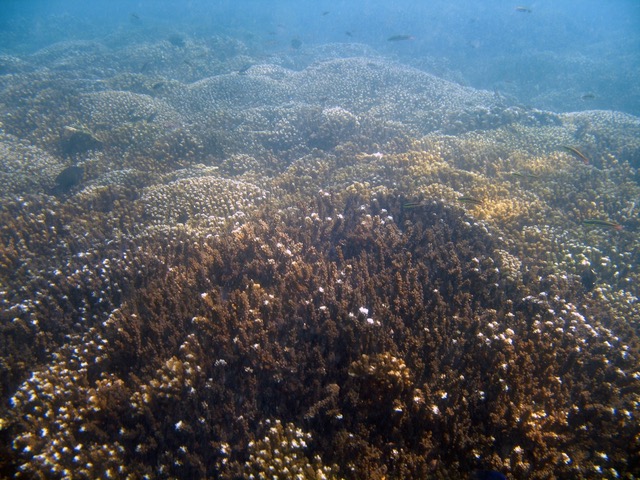


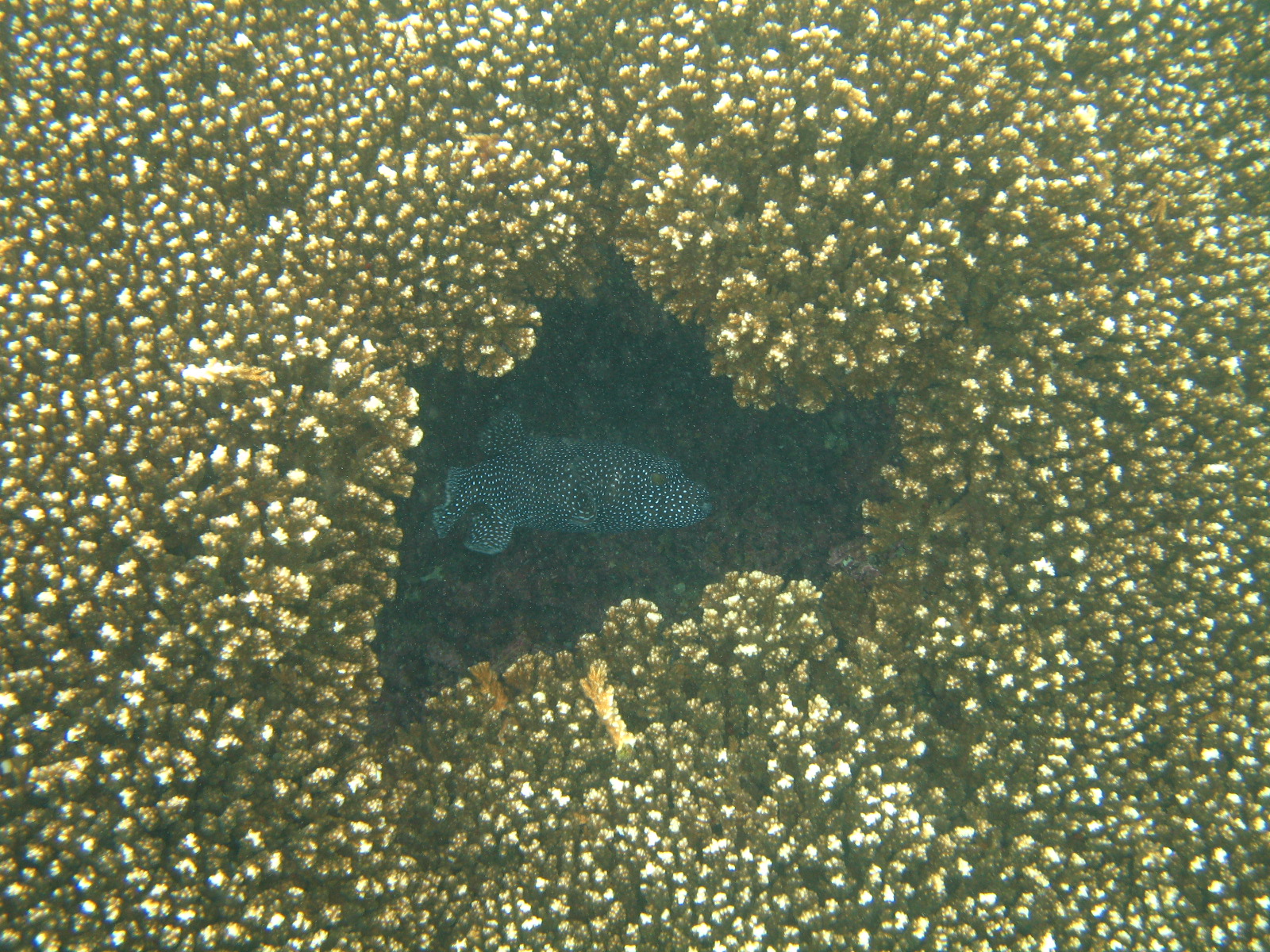

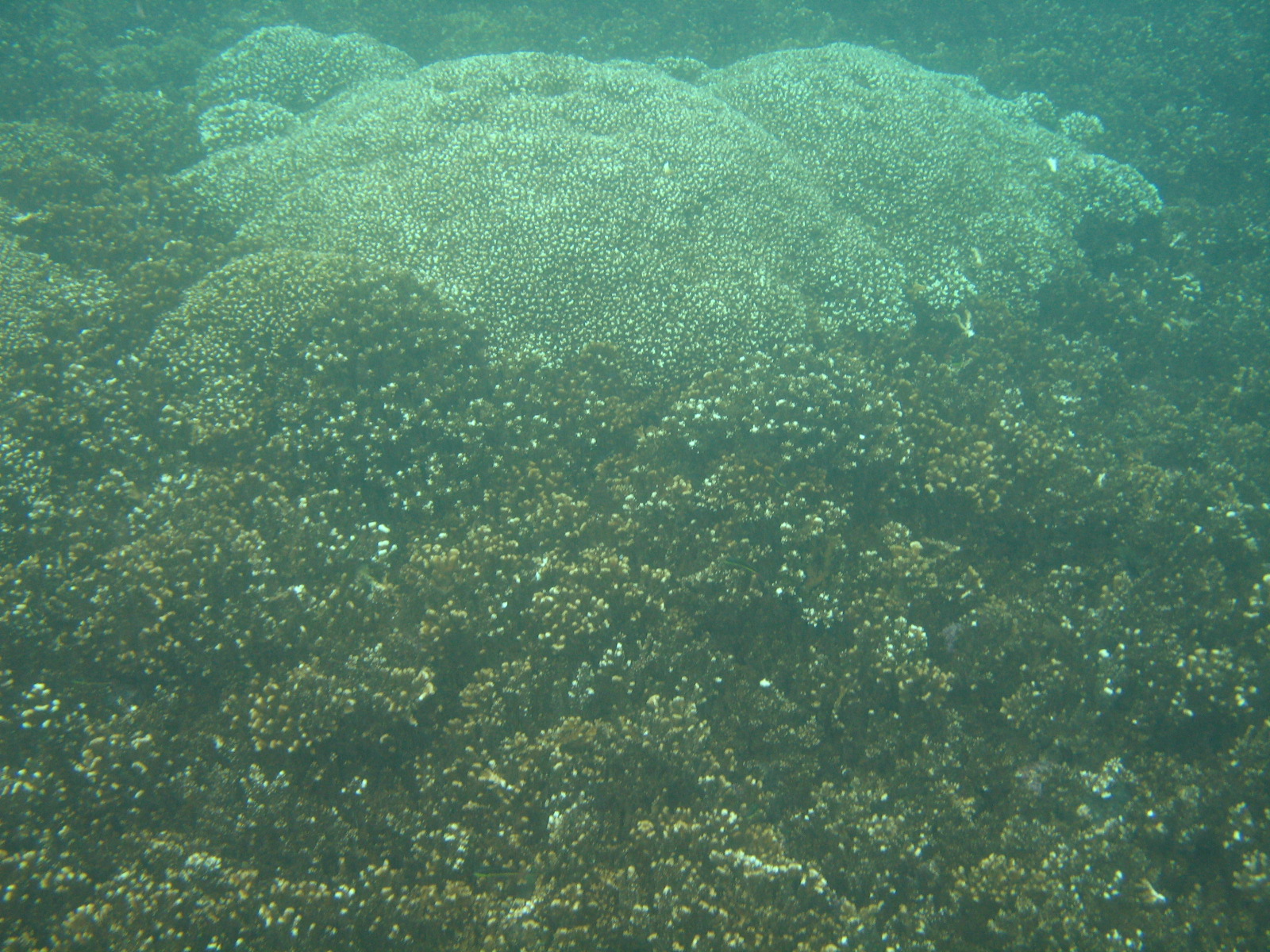

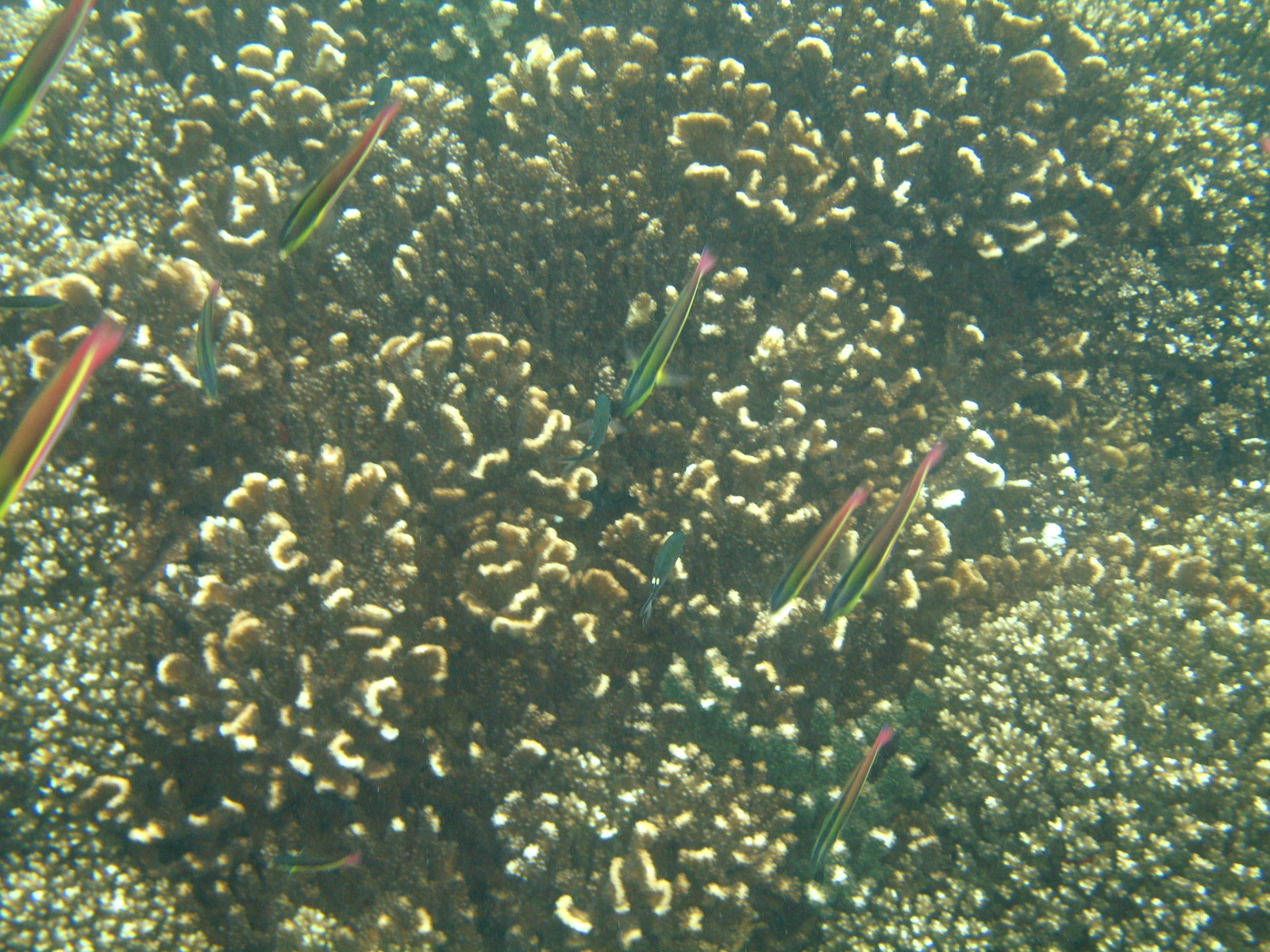

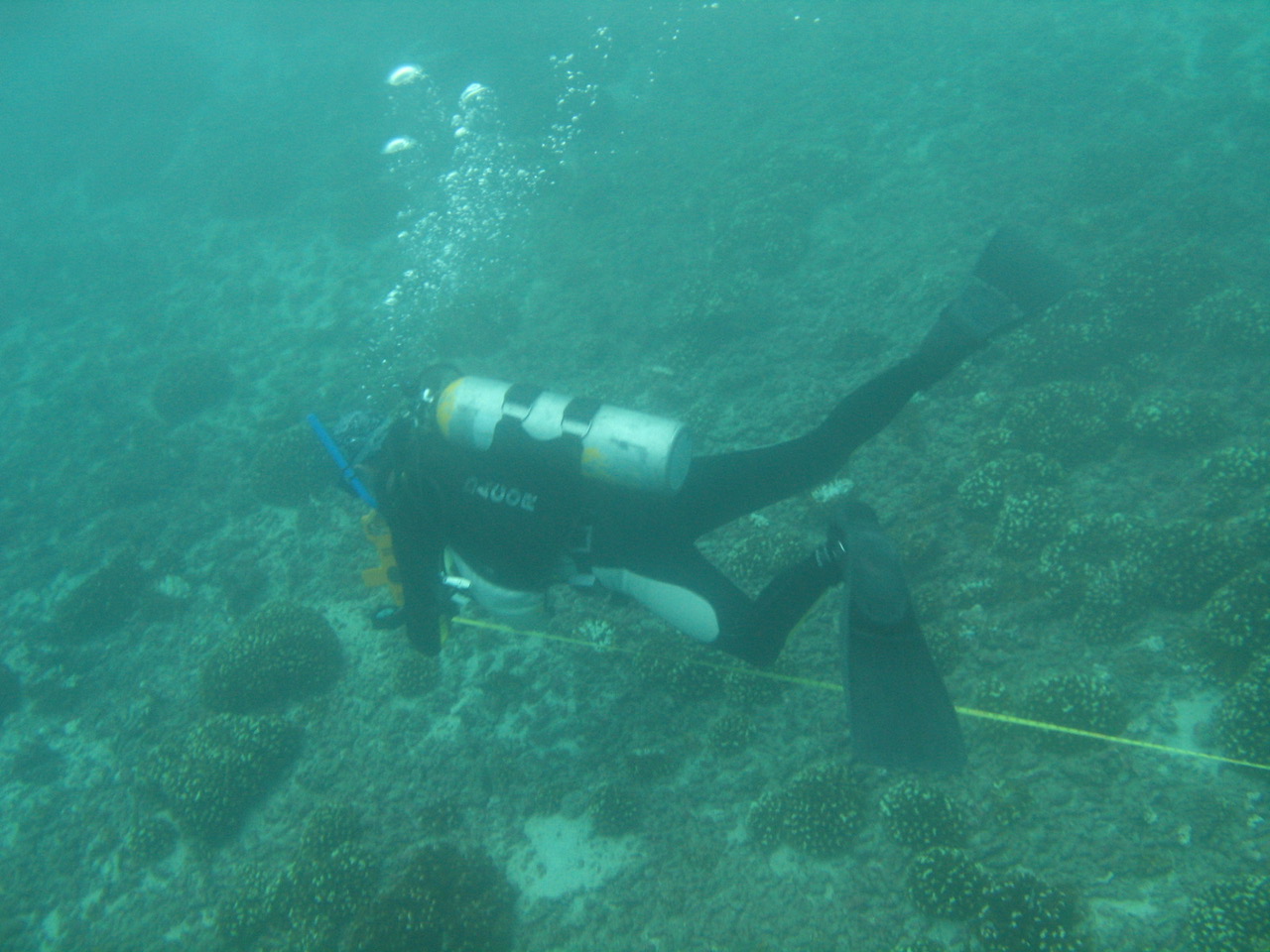


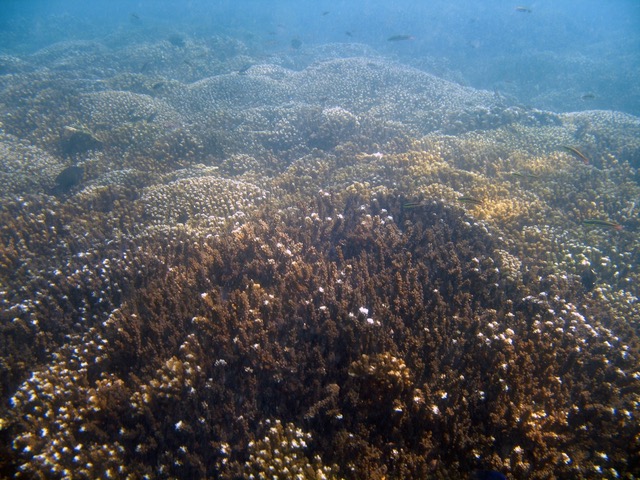

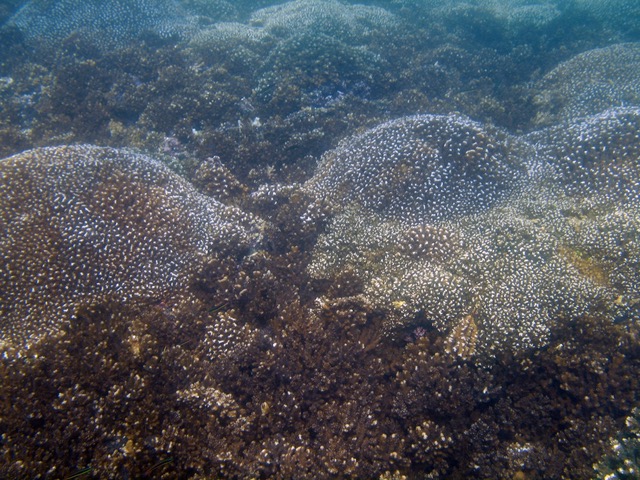

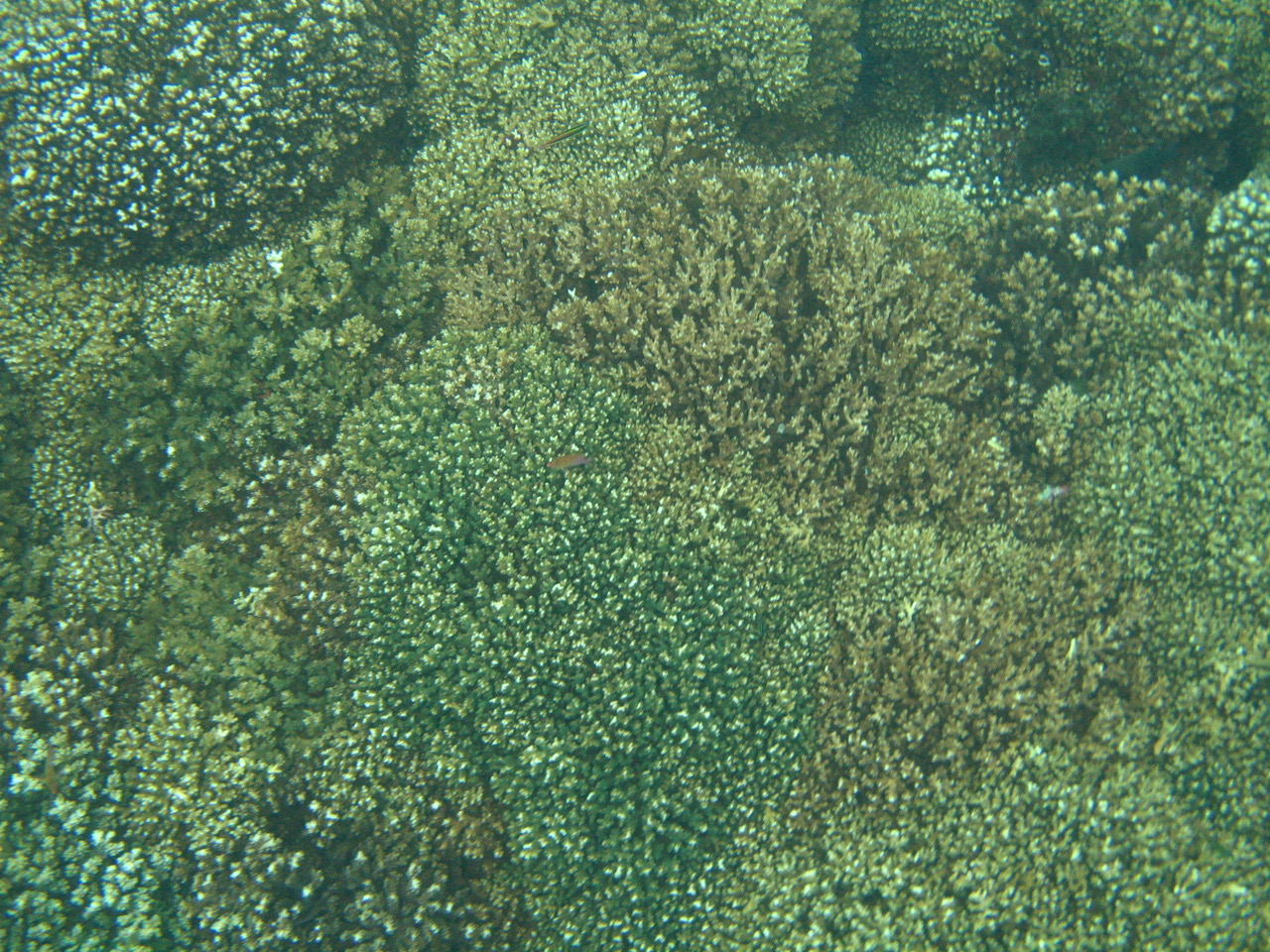

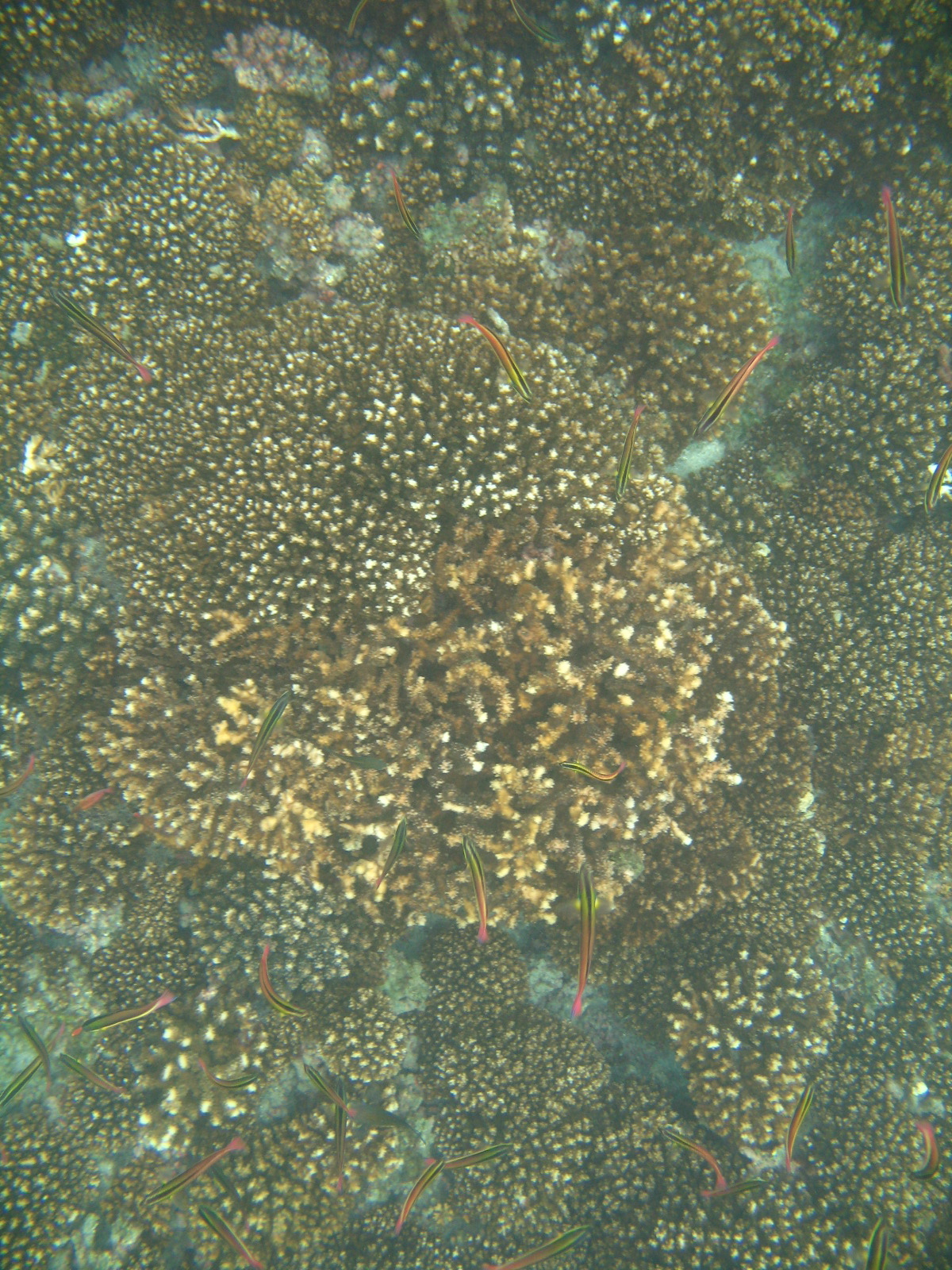


San Pedrito Reef post collapse, images from 2017


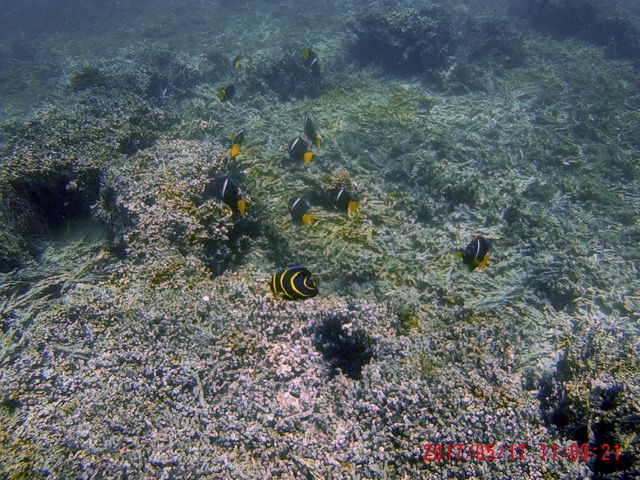


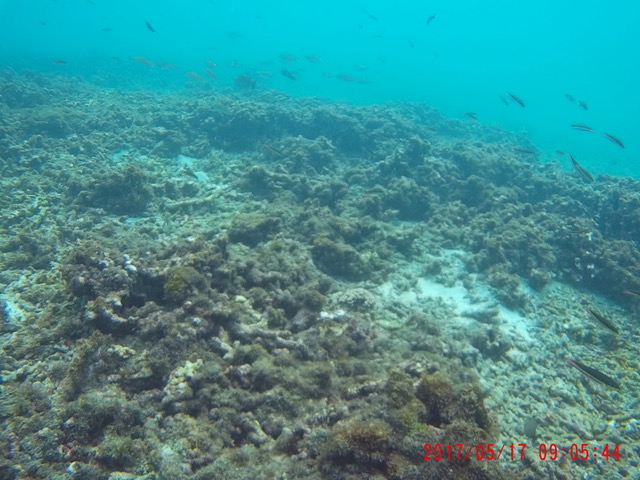

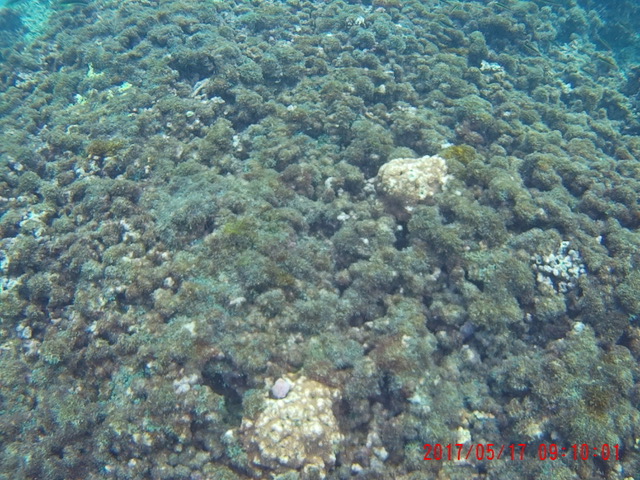

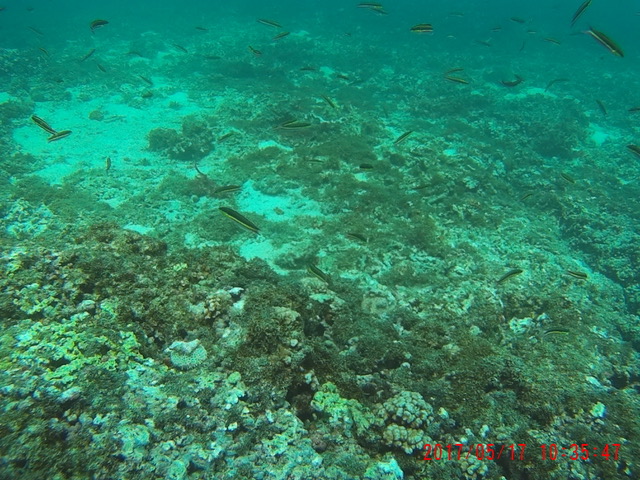


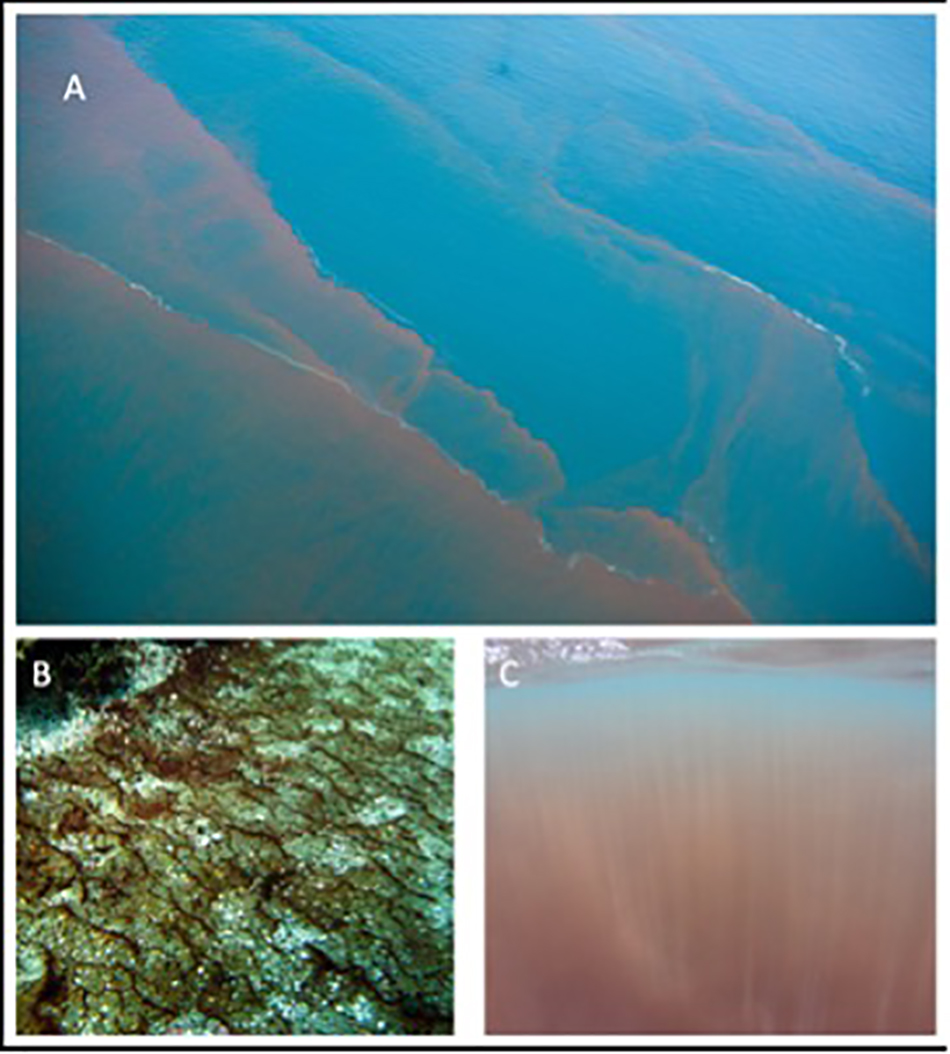


Harmful Algal Blooms (HAB) and cyanobacteria in Sector Marino; (a) aerial photo of HAB taken 24th February 2012 (ca. 1000m altitude) by Luciano Capelli, (b) carpet of cyanobacteria on a sand substrate, Sector Marino, taken May 2009 by Carlos Jimenez, (c) HAB in the water column at Golondrina taken 31^st^ March 2017.
